# The Pharmacological Effects and Health Benefits of *Platycodon grandiflorus*—A Medicine Food Homology Species

**DOI:** 10.3390/foods9020142

**Published:** 2020-01-31

**Authors:** Ming-Yue Ji, Agula Bo, Min Yang, Jin-Fan Xu, Lin-Lin Jiang, Bao-Chang Zhou, Min-Hui Li

**Affiliations:** 1 Baotou Medical College, Baotou 014060, Inner Mongolia, China; Jimingyue9@163.com (M.-Y.J.); agula372000@126.com (A.B.); yangmin_0406@aliyun.com (M.Y.); xjf0815@163.com (J.-F.X.); 2 Department of Pharmacy, Inner Mongolia Medical University, Hohhot 010110, Inner Mongolia, China; jianglinlin27@163.com (L.-L.J.); zbc373284882@163.com (B.-C.Z.); 3 Pharmaceutical Laboratory, Inner Mongolia Autonomous Region Academy of Chinese Medicine, Hohhot 010020, Inner Mongolia, China; 4 Inner Mongolia Key Laboratory of Characteristic Geoherbs Resources Protection and Utilization, Baotou Medical College, Baotou 014060, Inner Mongolia, China; 5 Guangxi Key Laboratory of Medicinal Resources Protection and Genetic Improvement, Guangxi Botanical Garden of Medicinal Plants, Nanning 530023, Jiangxi, China

**Keywords:** *Platycodon grandiflorus*, medicinal food, saponins, human health, applications

## Abstract

*Platycodon grandiflorus* is a widely used edible, traditional Chinese medicinal herb. It is rich in saponins, flavonoids, phenolic acids, and other compounds. It contains a large number of fatty acids such as linoleic acid (up to 63.24%), a variety of amino acids, vitamins, and multiple essential trace elements. *P. grandiflorus* has several biological applications, such as in hypotension, lipid reduction, atherosclerosis, inflammation, relieving cough and phlegm, promoting cholic acid secretion, and as an antioxidant. Further, *P. grandiflorus* is often used in the development of cold mixed vegetables, canned vegetables, preserved fruit, salted vegetables, and cosmetics in northeast China, South Korea, Japan, and Korea. In this paper, the active chemical components and the health benefits of *P. grandiflorus* have been reviewed, providing new ideas for the further development of nutraceutical products to prevent and manage chronic diseases.

## 1. Introduction

**Figure 1:**
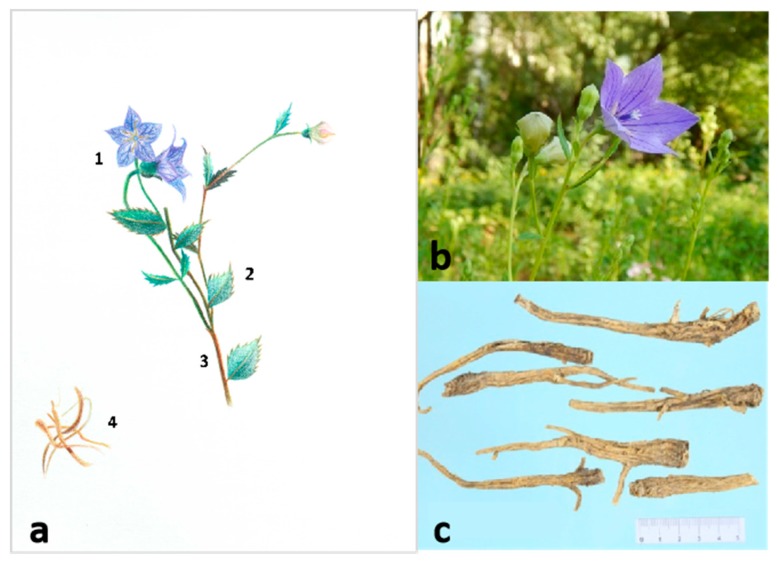
Images of *P. grandiflorus*. (**a**) Line drawing of *P. grandiflorus*: 1. flower; 2. leaf; 3. stem; 4. root. (**b**) Plant of *P. grandiflorus*. (**c**) The medicinal material of *P. grandiflorus*.

In recent years, with the gradual enhancement of public health awareness, healthy diets have been recognized as a significant and beneficial health factor. When people keep good healthy diet habits, they also enrich the varieties of food, and take the medicinal plants with therapeutic effect as food into daily life. This kind of “food” not only can satisfy hunger, but also has many functions, such as nutrition, health care, disease prevention, and treatment [1]. The food with this function is defined as a medicine food homology species. The theory of “medicine food homology” was formally put forward in the 1920s and 1930s, and its formation is a long process. *Platycodon grandiflorus* (Figure 1) is a perennial herb belonging to the family Campanulaceae, and it is a medicine food homology species. *P. grandiflorus* has been used as food and medicine for thousands of years in east Asia, such as China, Japan, and Korea. The description of *P. grandiflorus* was first recorded in *Shennong Bencao* in China. Later, it was documented in many other well-known medicinal works in other countries, including *Hanaoka Seishu* (Edo age of Japan, 1760–1835 A.D.) [2,3]. *P. grandiflorus* is rich in amino acids, plant fiber, vitamins, calcium, zinc, potassium, iron, and other trace elements essential in the human diet. It contains more than 16 amino acids, including 8 essential amino acids [4]. The tender seedlings and roots of *P. grandiflorus* have a broad market in Korea, South Korea, Japan, and northeast Chinese traditional wild vegetables [5]. The method of eating is to process *P. grandiflorus* into pickles, salads. Modern technology can be used for noodles, preserved fruits, and health drinks [6]. In addition, the flower of *P. grandiflorus* is blue, purple or white, and its shape is like a hanging clock, which has a very high ornamental value [4].

The chemical composition of *P. grandiflorus* was first studied by Japanese scholars in the early 20th century [7]. Further studies in modern pharmacology have shown that *P. grandiflorus* contains chemical compounds such as flavonoids, phenolic acids, triterpenoid saponins, polyacetylene, and sterols [8]. These are the main biological components that show significant antitussive, antitumor, antioxidation, anti-inflammatory, hypoglycemic, anti-obesity, and immune enhancement effects. Korean scholars have also found that the alcoholic extract of *P. grandiflorus* has a protective function in mitomycin-induced mutagenesis. *P. grandiflorus* can cause local tissue excitation, contact dermatitis, and hemolysis, and is an inhibitor of the central nervous system, which can reduce blood pressure. *P. grandiflorus* can also reduce tobacco toxicity and control the blood alcohol content in humans; it can thus be made into tobacco additives and alcohol absorption inhibitors [7]. Based on these properties, *P. grandiflorus* is often used in traditional Chinese medicine for respiratory system diseases [9]. In addition to these effects, platycodin D (PD), the main active compound extracted from *P. grandiflorus,* can inhibit lipase activity [10,11,12,13]. This property can be utilized in health foods to prevent and treat lipid metabolic disorders [14,15]. Therefore, *P. grandiflorus* can be used to treat various disorders. Most studies on *P. grandiflorus* report the medicinal aspects of the herb, while there are limited studies on medicine food homology.

As a medicine food homology species, *P. grandiflorus* is in great demand in the market. At present, the output in a normal year in China is 1 million kg, of which the export accounts for half. It is reported that 150 thousand kg of *P. grandiflorus* is needed annually in Japan [5]. *P. grandiflorus* as an export vegetable has become a new bright spot in increasing farmers’ income and its economic benefit is 2.5 times higher than as medicinal [16]. Many countries demand for *P. grandiflorus* increased stably, the export of fresh *P. grandiflorus* increased sharply; the demand exceeds the supply, the price rises greatly, therefore, *P. grandiflorus* has huge development value and good development prospect.

In this review, the active chemical components and pharmacological activities of *P. grandiflorus* have been summarized based on the literature review. In addition to medicine, alternative applications of *P. grandiflorus* were introduced to provide a new understanding in the homology of medicine and food, with the ultimate goal of using this herb as a naturally-derived therapeutic option.

## 2. Bioactive Components

### 2.1. Saponins

Saponin is a type of glycoside whose aglycone is a triterpenoid or spirosterol. Triterpenoid saponins are abundant in *P. grandiflorus* [17,18]. They are the main active component characteristic to *P. grandiflorus,* and are olefin-type pentacyclic triene derivatives. According to the parent nucleus of the saponins, they can be divided into platycodic acid, platycogenic acid, and polygalacic acid [19,20,21]. According to the Pharmacopoeia of the People’s Republic of China, the saponin content should not be less than 6.0% by gravimetric method in order to control the quality of medicinal materials [22]. At present, 75 triterpenoid glycosides have been isolated and identified from *P. grandiflorus*. Among them, platycodin A is considered to be the main saponin of *P. grandiflorus*.

**Figure 2:**
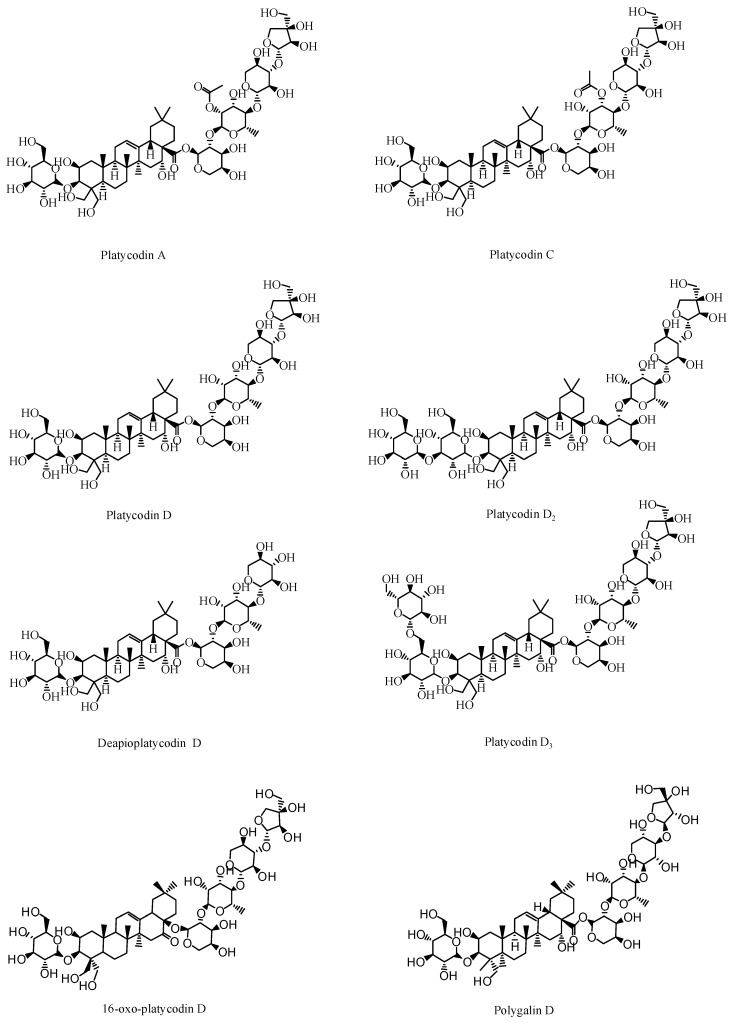
Active triterpenoid saponins in *P. grandiflorus.*

Studies have also confirmed that PD is the main active compound in the extract of *P. grandiflorus* [23]. Guo [24] determined that the PD was present in all parts of the *P. grandiflorus* herb. However, the content of PD in the upper portion of roots and leaves was slightly lower than that of the main root, and the content of PD in the fibrous roots and root bark of the *P. grandiflorus* was higher than that in the aerial parts. PD is both medicinal and nutritional and has high anti-tussive [25], anti-obesity [26], anti-fibrosis [27], anti-inflammatory, and anti-tumor effects [28]. In vitro experiments showed that platycodin D_3_ could eliminate phlegm and showed anti-inflammatory activity. In addition, platycodin D_2_, platycodin D_3_, and PD have significant antitumor activity [29,30,31]. Platycodin A, platycodin C, deapioplatycodin D, and 16-oxo-PD have been shown to have anti-obesity activities [32,33,34,35]. The saponins structures are shown in Figure 2.

### 2.2. Flavonoids

**Figure 3:**
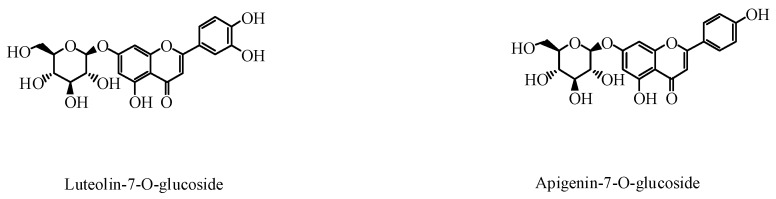
Active flavonoids in *P. grandiflorus.*

Flavonoids mainly exist in the upper portion of *P. grandiflorus* above the soil, mainly comprising of flavonoids, dihydroflavonoids, and flavonoid glycosides. At present, 11 flavonoids have been isolated and identified from *P. grandiflorus* [36]. It has been shown that six different flavonoids were obtained from the seeds and the flowers of *P. grandiflorus*, while 3 compounds were isolated from the aboveground part of *P. grandiflorus* grown in Poland [37]. Of these flavonoids, luteolin-7-*O*-glucoside and apigenin-7-*O*-glucoside exhibit strong antioxidant activity [37,38]. The structures of the flavonoids are shown in Figure 3, and the other nine flavonoids isolated from *P. grandiflorus* are listed in Table 1.

### 2.3. Other Components

In addition to saponins and flavonoids, *P. grandiflorus* contains other compounds, such as phenolic acids, polyacetylene, sterols, and amino acids [40,41,42,43,44]. Phenolic acids are abundant in the roots and aboveground parts of *P. grandiflorus*; 14 kinds of antioxidant phenolic compounds have been isolated from the *P. grandiflorus* extract. Five polyacetylene compounds have been obtained from *P. grandiflorus*, which is an important criterion for the classification of *P. grandiflorus* [45]. Lobetyol has been found to have an anti-tumor effect.

**Figure 4:**
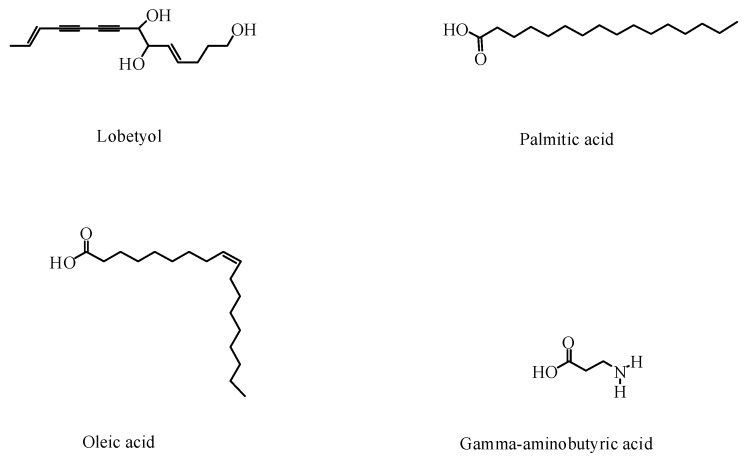
Other active components in *P. grandiflorus.*

Macromolecules have also been identified in P. grandiflorus. A study showed that P. grandiflorus contained 18 amino acids [32]. Among these, gamma-aminobutyric acid is an essential neurotransmitter chemical in the brain’s energy metabolism. In addition, the root of *P. grandiflorus* contains fatty acids, which accounted for 88.28% of the total lipids [46]. Inulin, grandoside, and polysaccharides have also been isolated from *P. grandiflorus* [47]. Studies have shown that the polysaccharides of *P. grandiflorus* have strong antioxidant activity [48]. Although the contents of bioactive components of *P. grandiflorus* were different, they all had high efficiency and low toxicity, which provided a scientific basis for characterizing its pharmacological activities. The structures of the other components are shown in Figure 4, and the other non-active components are listed in Table 2.

## 3. Pharmacological Actions

**Figure 5:**
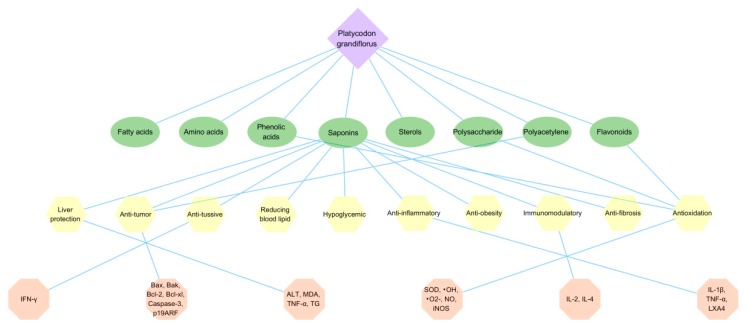
The pharmacological activity mechanism of *P. grandiflorus*. The green ovals represent some activity constituents, yellow polygons represent the common pharmacological activities of *P. grandiflorus*, while represented enzymes and signaling pathways are illustrated by pink polygons. Abbreviations here represent the same meaning as in the body text.

*P. grandiflorus* has high edible and medicinal value, contains a variety of active ingredients beneficial to the human body, has relieving cough and asthma activities, anti-tumor, anti-inflammatory and antibacterial, antioxidation, hypoglycemic, liver protection, improves human immunity and other broad pharmacological activities, has good clinical application value and research potential. The main pharmacological activities and the underlying mechanisms are shown in Figure 5.

### 3.1. Relieving Cough and Asthma Activities

*P. grandiflorus* exhibits strong antitussive, expectorant, and antiasthmatic effects. In an *in vivo* asthma study [50], guinea pigs were randomly divided into five groups: the normal control group, the asthma model group, the dexamethasone group, the *P. grandiflorus* root extract low-dose group, and the *P. grandiflorus* root extract high-dose group. Except for the normal control group, the asthma model was established by ovalbumin in other groups. After successful modeling, the effects of *P. grandiflorus* extract on the levels of serum-related indexes in experimental bronchial asthmatic guinea pigs were observed. The results showed that there was no significant difference in serum-related indexes between the high-dose group and the normal control group (*p* > 0.05). This indicates that the high-dose of the *P. grandiflorus* extract can effectively prolong the latent period of asthma and significantly reduce the generation and release of oxygen free radicals. The extract was also found to simultaneously promote IFN-y secretion, thereby indirectly playing the role of regulating the Thl/Th2 balance, and promoting the release of lipoxin A4 (LXA4). The LXA4 in the body is adjusted to exert a wide anti-inflammatory and dissipation effect. Therefore, a high-dose extract treatment may be suitable for clinical use in asthma patients.

In another study [51], several animal models were used including chronic bronchitis in mice, guinea pigs with histamine-induced asthma, citric acid-induced cough in guinea pigs, the effects of carrageenin and cotton ball granuloma inflammation in rats. Different doses of platycodin were administered to detect several outcomes, including the number of cells in the alveolar lavage fluid of the slow-branch mice, the histamine-induced asthma reaction, the anti-cough response to the acorn acid and the excretion of the respiratory tract phenol red, the swelling of the foot of the rats, and the weight of the granuloma of the cotton ball. The results of this study suggested that the total number of cells in the alveolar lavage fluid and the number of neutrophils in the lung tissue were significantly lower than that of the control group, while the proportion of the lymphocytes and the macrophages increased and the latent period of the antitussive and cough was prolonged. In addition, cough and asthma was decreased, and the amount of phenol red excretion in the respiratory tract was increased with platycodin administration. This suggested that platycodin has significant antitussive, antiasthmatic, and expectorant effects, and is likely a beneficial for the treatment of chronic bronchitis.

Platycodin can inhibit the activity of nuclear factor kappa B (NF-κB) and downregulate the expression of mucin 5, subtypes A and C (MUC5AC) protein in aldehyde-induced lung cancer cells in a concentration-dependent fashion. This mechanism may be related to the inhibition of NF-κB activation by regulating reactive oxygen species (ROS)—protein kinase C (PKCS)—mitogen-activated protein kinase (MAPK) signaling pathway [52]. PD and platycodin D_3_ can also increase the release of respiratory mucin in rats and hamsters. Specifically, the dose of 20 µg/mL platycodin D_3_ could effectively promote the release of mucin in rats, and its effect was better than that of the positive control ATP and ambroxol at 200 µg/mL [53].

### 3.2. Anti-Tumor Activity

Studies have shown that PD, platycodin D_2_, and deapioplatycodin D have significant inhibitory effects on the proliferation of A549 (non-small cell lung), SK-OV-3 (ovary), SK-MEL-2 (melanoma), XF498 (central nerve system), and HCT-15 (colon) cell lines in vitro [28]. Kim et al. [54] studied the mechanism of PD induced human leukemia cells (U 937, THP-I, and K 562 cells) for proliferation and cell death. The mechanisms of cell apoptosis were investigated by evaluating cell growth and caspase-3 activity. The effects of different concentrations of PD on synchronous leukemia cells were induced by downregulating Cdc 2/cyclinB-1 and upregulating wee1 expression, resulting in mitotic arrest and endoreduplication, and upregulating CDK-2 protein by downregulating p21. The authors also studied the induction of polyploidy by microtubule polymerization. Their results showed that PD could significantly induce microtubule polymerization in leukemia cells. It revealed that the direct induction of microtubule polymerization in vitro required a high concentration of PD (>200 M). Finally, PD exposure induced apoptosis of U 937 cells by caspase-3 dependent PARP and laminin A. It is therefore logical to assume that the main anti-leukemia activity of PD is to induce internal replication and mitosis, which is caused by the kinetics of compression of the spindle microtubules and the promotion of apoptosis of leukemia cells.

Platycodin D can induce apoptosis in a variety of cancer cells. Yu et al. [55] found that PD activated apoptosis signal regulated kinase 1 (ASK 1) through phosphorylation of threonine ASK 1 and dephosphorylation of serine ASK 1. Moreover, PD induced the activation of endoplasmic reticulum (ER) stress response. The results showed that PD treatment could induce phosphorylation of PKR-like ER kinase (Perk) and eukaryotic initiation factor 2 α (ElF 2α). The expression of glucose regulated protein 78/immunoglobulin heavy chain binding protein (GRP 78/Bip) and CCAAT/enhancer binding protein homologous protein/growth block and DNA damage induced gene 153 (CHOP/GADD 153) were inhibited by N-acetyl-L-cysteine and activated by caspase-4. In addition, the stress responses of ASK 1 and ER induced by PD were also inhibited by N-acetyl-L-cysteine. These results suggest that ROS play a key role in activating ASK 1 and ER stress in PD-treated cancer cells.

Aside from platycodin, *P. grandiflorus* polysaccharides can significantly inhibit the tumor growth of U14 cervical cancer in mice, induce apoptosis of U14 tumor cells, increase the expression of P19ARF and Bax protein, and decrease the expression of mutant p53 protein. It is speculated that *P. grandiflorus* polysaccharides can have an anti-tumor effect by regulating the expression of related genes to promote the apoptosis of tumor cells [56].

### 3.3. Antioxidation Activity

In addition to antitussive and anti-tumor effects of *P. grandiflorus,* antioxidant effects have also been observed. To this end, the effects of *P. grandiflorus* saponins on the activity of antioxidant enzymes and the concentration of free radicals in lung tissues of mice with chronic bronchitis have been studied. Long-term smoking plus ammonia spray was used to establish chronic bronchitis and superoxide dismutase (SOD) activity of antioxidant enzymes in mice. The authors found that the concentration of free radicals in the lung tissue correspondingly increased, combined with increased activity of iNOS and chronic bronchitis. *P. grandiflorus* saponins significantly increased the activity of the antioxidant enzyme SOD and reduced the activity of the superoxide anion (•O_2_), hydroxyl radical (•OH), hydrogen peroxide (H_2_O_2_), nitric oxide (NO), and other free radicals as well as iNOS. There was an obvious dose-activity relationship, which significantly improved the oxidative stress injury [57].

Gu et al. [58] took H_2_O_2_-induced PC12 cells as a model of cell oxidative damage. Compared with the model group, *P. grandiflorus* polysaccharide treatment group reduced lactate dehydrogenase (LDH), Malondialdehyde (MDA), and ROS content and enhanced SOD and glutathione peroxidase (GSH-Px) activity in a statistically significant fashion (*p* < 0.05–0.01). In addition, *P. grandiflorus* polysaccharide inhibited the expression of NOX_2_, p22phox, and Rac proteins. The results confirmed that *P. grandiflorus* polysaccharide had a protective effect on H_2_O_2_-induced PC12 cells and could reduce apoptosis. This mechanism may be related to the inhibition of NOX_2_ overexpression.

Wang et al. [59] used oxidized low-density lipoprotein (OXLDL) to induce human umbilical vein endothelial cells (HUVECs) to establish its oxidation model. After treatment with different concentrations of the total saponin of *P. grandiflorus*, NO in the culture solution was measured. The level of MDA, and the expression of vascular cell adhesion molecule-1 (VCAM-1) and intercellular cell adhesion molecule-1 (ICAM-1) were used to observe the effects of the total saponin of *P. grandiflorus* on the oxidative damage of oxidized low-density lipoprotein-induced endothelial cells. The results showed that PD was able to significantly reduce the levels of NO and MDA in the cells, reduce the expression of VCAM-1 and ICAM-1 and the adhesion of monocytes and endothelial cells. The authors proposed that the total saponin of *P. grandiflorus* could be a new effective drug with potential antioxidant, lipid-lowering, and anti-atherosclerosis effects.

### 3.4. Anti-Inflammatory and Antibacterial Activities

Jang et al. [60] studied the anti-inflammatory effect of saponins isolated from *P. grandiflorus* on the production of inflammatory mediators and cytokines in the microglia of BV2 mice stimulated by lipopolysaccharide (LPS). Elevated NO, prostaglandin E (PGE2), and proinflammatory cytokines were detected in BV2 microglia after LPS stimulation. However, *P. grandiflorus* significantly inhibited the excessive production of NO, PGE2, and pro-inflammatory cytokines, including interleukin-1*β* (IL-1*β*) and TNF-α in a concentration dependent manner, without causing any cytotoxic effects. In addition, *P. grandiflorus* inhibited NF-κB translocation and LPS-induced phosphorylation of AKT and MAPKs. The results suggest that the inhibition of *P. grandiflorus* on the inflammatory response stimulated by LPS in BV2 microglia is related to the inhibition of the activation of NF-κB and the PI3K/AKT and MAPK signaling pathways. Thus, these findings suggest that *P. grandiflorus* may play a role in the treatment of neurodegenerative diseases by inhibiting the inflammatory response of activated microglia.

Zhu et al. [61] studied the effect of PD on the adhesion of *Candida albicans* to oral mucosal epithelial cells. With the increase in the PD concentration of the *P. grandiflorus* saponin, the change in *C. albicans* from spore phase to mycelium phase was gradually reduced, and the number of adhesive spores and their vitality gradually decreased. Moreover, mRNA levels of IL-8 and human *β*-defensin (HBD) in supernatant fluid-2 protein and HBD-2 KB cells were gradually reduced, indicating that the characteristics of saponin D reduce *C. albicans* infection of the oral mucosa.

In a separate study [62], a model of chronic bronchitis was established by smoking and ammonia inhalation in mice. Immunohistochemical examination showed that the expression of IL-1*β* and TNF-*α* in the lung cells of the model group was significantly higher than that of the normal control group. After 30 days of continuous administration, the expression of IL-1*β* and TNF-*α* in lung cells in each treatment group was significantly reduced compared to that in the model group (*p* < 0.05, *p* < 0.01). Western blotting revealed significantly increased levels of IL-1*β* and TNF-*α* in lung tissue cells in the model group, compared with normal controls (*p* < 0.01). However, after 30 days of continuous administration, the expression levels of IL-1*β* and TNF-*α* in the lung cells of the mice in each treatment group were significantly decreased, with a strong dose-response relationship. The results showed that *P. grandiflorus* saponin had a significant inhibitory effect on the expression of inflammatory cytokines IL-1*β* and TNF-*α* in the lung tissues of mice with chronic bronchitis. It was speculated that the mechanism of action may have been through inhibiting the production of inflammatory cytokines and free radicals in lung tissues to achieve anti-inflammatory effects.

### 3.5. Hypoglycemic Activity

Various studies have demonstrated that *P. grandiflorus* shows anti-diabetic activity. The hypoglycemic effect of *P. grandiflorus* extract on diabetic institute of cancer research (ICR) mice was evaluated. The results showed that *P. grandiflorus* ethanol extract could relieve hyperglycemia induced by glucose stimulation. Compared with the model control group, *P. grandiflorus* enhanced the hypoglycemic effect of exogenous insulin without stimulating insulin secretion, suggesting that the insulin sensitivity of diabetic mice increased [63]. Chen et al. [64] treated streptozotocin (STZ)-induced impaired glucose tolerance (IGT) mice with increasing doses of *P. grandiflorus* and found that it had significant inhibitory effect on the activity of α-glucosidase in vitro and in vivo. There was a significant reduction in the blood glucose level of the STZ-IGT mice after oral administration of *P. grandiflorus*. The ethanol extract of *P. grandiflorus* could significantly reduce the blood glucose level in IGT mice at 30 min after meals. This suggested that high doses of ethanol extract of *P. grandiflorus* can significantly decrease blood glucose in IGT mice. It also indicates that reduced blood glucose caused by *P. grandiflorus* is related to the inhibition of α-glucosidase activity.

Qiao et al. [65] believed that for diabetic rats, *P. grandiflorus* can significantly reduce the water intake, food intake, and urine output. The fasting blood glucose in groups administered low, medium, and high doses of *P. grandiflorus* was significantly lower than that in the model group (*p* < 0.05 or *p* < 0.01), and the fasting insulin level, insulin sensitivity index, and glucose tolerance were significantly increased (*p* < 0.05 or *p* < 0.01). *P. grandiflorus* polysaccharides can also increase the activity of superoxide dismutase in liver tissue and decrease the content of malondialdehyde (*p* < 0.05 or *p* < 0.01), indicating that *P. grandiflorus* has a hypoglycemic effect. The mechanism may be to improve fasting insulin levels and antioxidant capacity.

Zheng et al. [66] reported that ethanol extract of *P. grandiflorus* root significantly reduced blood glucose levels in streptozotocin (STZ) diabetic mice, and reduced oral glucose tolerance after 30 min. Though blood glucose levels decreased significantly after combined treatment of STZ diabetic mice, the ethanol extract of *P. grandiflorus* did not affect plasma insulin levels.

### 3.6. Liver Protection Activity

*P. grandiflorus* has a therapeutic effect on a variety of drug-induced liver injury models. Khanal et al. [67] studied the protective effects of saponins isolated from the roots of *P. grandiflorus* (Changkil saponins: CKS) on liver injury in mice induced by ethanol. The results showed that levels of serum aminotransferase (ALT) and liver TNF-*α* increased significantly in the model group, the production of MDA increased, the amount of triglyceride (TG) increased significantly, and the level of GSH in liver tissue decreased significantly, indicating that ethanol induced liver injury in mice. Compared with the model group, the levels of serum ALT, TNF-*α*, MDA, and TG in the liver of the experimental group were significantly increased, which showed a dose-dependent relationship. Microscopical observation showed that the mice in the model group showed morphological changes such as liver tissue deformation, but on pretreatment with CKS, these changes were significantly inhibited. The above results indicate that CKS may block CYP2E1-mediated ethanol bioactivity and scavenging free-radical inhibition of ethanol-induced liver injury.

Luan et al. [68] observed the effects of total saponins of *P. grandiflorus* on blood glucose, blood lipids, and liver function of type 2 diabetic rats established by tail vein injection of STZ (15 mg/kg) and high glucose and high fat diet for 4 weeks. Total saponins of *P. grandiflorus* (200 mg/kg) for 18 weeks was able to reduce blood sugar, serum cholesterol, triglyceride, low-density lipoprotein levels, and increase serum high-density lipoprotein levels, and improve liver function, thereby reducing type 2 diabetic liver damage in rats.

Hou et al. [69] proved that *P. grandiflorus* and Na_2_SeO_3_ as raw materials have different degrees of protective effects on liver injury induced by CCl_4_ in mice, and the best liver-protecting effect is the high-dose nano-selenium *P. grandiflorus* polysaccharide complex group.

### 3.7. Other Activities

In addition to the above pharmacological activities, *P. grandiflorus* also has anti-obesity, immune-modulating activity, anti-fatigue, anti-pulmonary damage, and other biological activities.

Zheng et al. [70] found that the water extract of *P. grandiflorus* inhibits the activity of pancreatic lipase, thereby inhibiting the hydrolysis of trioleate and lecithin mixed microparticles, and can reduce the triacyl content in the plasma of rats fed high corn oil. These results suggest that the water extract of Campanulaceae can inhibit the absorption of food fat by the small intestine. Wang et al. [71] found that PD in *P. grandiflorus* showed immunomodulatory activity, stimulating spleen lymphocytes to enhance their proliferative capacity and inducing the secretion of IL-2 and IL-4, and increasing the ratio of CD4^+^/CD8^+^. It suggested that PD could promote the development of splenic lymphocytes from G0/G1 phase to S phase. Yu et al. [72] reported treatment with high, medium, and low doses of an ethanol extract of *P. grandiflorus* could significantly protect against fatigue in mice. Extracts prolonged the mouse climbing rod and swimming time, and significantly increased the reserve of liver glycogen and muscle glycogen after exercise, thus achieving the anti-fatigue effect. Yao et al. [73] also reported that total saponins of *P. grandiflorus* may significantly reduce the inflammatory lesions of lung tissue induced by PM2.5 (PM with aerodynamic diameters ≤2.5 μm) in rats by regulating cytokine and down-regulating the expression of TGF-*β* and inhibiting the development of fibrosis, which results in the protection and repair on the lung injury of rats caused by PM2.5.

## 4. Application

### 4.1. Patent Release of P. grandiflorus

**Figure 6:**
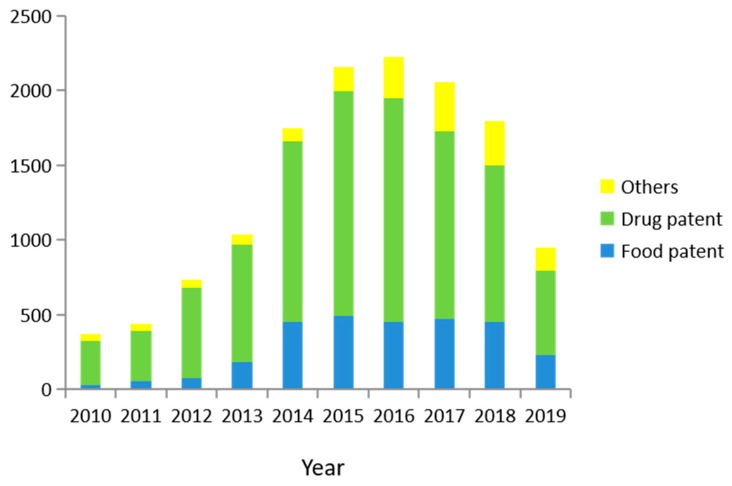
Patent statistics of *P. grandiflorus* from 2010 to 2019.

With the improvement of people’s living standards and public health awareness, increased attention has been paid to the development of *P. grandiflorus.* There will be increased space for the development of *P. grandiflorus* based on the numerous findings identifying its pharmacological activities. According to the database of Baiten, using *P. grandiflorus* as the key term to search, a total of 15,712 patents were retrieved, including 15,676 Chinese patents. In addition, a total of 37 patents were retrieved from the World Intellectual Property Organization. Figure 6 shows the publication of patents in the last ten years. However, the results show that the number of *P. grandiflorus* patents have declined since 2016. This is likely due to the shortage of *P. grandiflorus.* The decline is likely to continue in the coming years.

Through additional analysis, it was found that most of the patents were related to drugs, food, necessities of life, and agriculture. Among them, the number of patents for drugs and food accounted for a larger proportion. Patent statistics show that drugs and foods account for 72.09% and 19.98% of the total patents, respectively. The number of drug patents declined year by year from 2015, while the number of food patents remained relatively stable. With the improvement of public health care awareness, more people focus on the development of health food, not only in the development of drugs. At present, the health food field mainly includes tea, drinks, cakes, noodles, and preserved fruit from *P. grandiflorus*. Among them, the tea from *P. grandiflorus* has the most patents, at 555. In general, *P. grandiflorus* patents have shown a decreasing trend in recent years. Therefore, research on *P. grandiflorus* still needs attention and improvement. The development of medicinal and health care products will undoubtedly be the focus of future research [74]. In the near future, the application of the homologous medicinal herbs will continue to increase.

### 4.2. Food Application

*P. grandiflorus* is highly edible and is often used in food in North Korea, South Korea, Japan, and China (Yanbian region). The tender seedlings and roots of *P. grandiflorus* are edible and contain high levels of starch, proteins, and vitamins, and more than 16 kinds of amino acids, including 8 kinds of amino acids necessary for human body. The root of *P. grandiflorus* contains 61.20% sugar and 2.44 mg vitamin B_2_ per 100 g. The content of starch, protein, and fiber is 14.00%, 0.19%, and 3.19% in fresh vegetables, respectively. For every 100 g of fresh vegetables, the content of carotene is 8.8 mg and vitamin B_1_ is 3.8 mg, which can be made into noodles and other delicious dishes [4]. Fresh roots of *P. grandiflorus* not only preserve its medicinal value, but also the taste and color.

Liu et al. published an invention on nutrient noodles of *P. grandiflorus* [75]. Noodle additives such as wheat oligopeptides and zinc rich arachis oil enhance the taste of the noodles and enhance immunity, thereby promoting the growth and development of children. They are also expected to contribute to anti-oxidation, anti-aging, and improving life expectancy. A *P. grandiflorus* cake with health care functions was invented by Liang et al. [76]. They used processes including raw material handling, mixing, molding, baking, cooling, and packaging to produce the cake. The product is soft, sweet, and delicious, with a flavor of *P. grandiflorus*. This product has a high nutritional value, is low in calories, and is a low-fat green food which integrates nutrition and health care values. In addition, a clear and transparent medicinal liquor was developed, with a unique flavor of *P. grandiflorus*, which retains the efficacy of the traditional Chinese medicine to improve the curative effects of the active ingredients. Tao published the method of making medicinal wine of *P. grandiflorus* [77], a type of health wine that is low in calories, sugar, and fat. It has the effect of relieving cough and resolving phlegm. People can combine the functions of *P. grandiflorus* as a food and medicine, to integrate a medicated diet that aims to keep people fit.

### 4.3. Clinical Application

In traditional Chinese medicine, the rhizome of *P. grandiflorus* has been widely used. *P. grandiflorus* is mainly used as an expectorant, which has significant effects in treating cough, phlegm, chest tightness, sore throats, and other disorders. It has been documented in many medical books, such as *Shennong Bencao* (Han Dynasty), *Bencao Yanyi* (Song Dynasty, 1116 A.D.), *Bencao Gangmu* (Ming Dynasty, 1590 A.D.), and others. Fortunately, in the Ming Dynasty, Li Shizhen made a systematic summary of *P. grandiflorus* (Jiegeng in Chinese) in *Bencao Gangmu*. In this book, the roots of *P. grandiflorus* could be used to treat cough with chest distress, pulmonary abscesses, and other diseases. In addition, *P. grandiflorus* can usually be used in combination with other herbs, such as *Pinellia ternata*, *Lycium chinense* Mill., *Glycyrrhiza uralensis* Fisch., and others that can increase its therapeutic effect. In the current clinical practice of Chinese medicine, the effects of *P. grandiflorus* are obvious, and with few side effects, its use is very popular. It is widely used as a medicinal compound and Chinese patent medicine in modern clinical medicine, mainly for the treatment of cough, bronchitis, faucitis, and bronchial asthma [78]. In addition, using herbal products as alternative medicines could avoid surgical injury and could also be useful in Western medicine. Researchers have found the advantages of combining Chinese and Western medicine, that can be synergistic and reduce toxicity and side effects, by decreasing drug dosage and extending the adaptive range [79].

Patients with early breast cancer often receive anthracycline-based chemotherapy. However, anthracycline can cause dose-dependent cardiotoxicity. As a traditional Chinese medicine, *P. grandiflorus* has been used for thousands of years to treat cardiovascular diseases. In one study, researchers evaluated the cardioprotective effects and safety of *P. grandiflorus* in patients with early breast cancer receiving anthracycline-based chemotherapy. *P. grandiflorus* may have the potential to prevent anthracycline-induced cardiotoxicity, and also has the advantage of being more affordable [80].

Kikyo-to (KKT) is a formula combination of *Glycyrrhiza uralensis* Fisch. root and *P. grandiflorus* root extracts, which is used for relieving sore throats associated with acute upper respiratory tract infection (URTI) in Japan. This formula is prescribed in primary care. In one study, the therapeutic effect of KKT was sufficient to significantly reduce sore throat, and no side effects were observed [81]. Kikyo-to is a part of Sho-saiko-to-ka-kikyo-sekko. Sho-saiko-to-ka-kikyo-sekko is composed of 9 herbs (gypsum, *Bupleurum* root, *Pinellia* tuber, *Scutellaria* root, *Platycodon* root, jujube fruit, ginseng root, *Glycyrrhiza* root, and ginger rhizome). In some cases, this fixed combination can cure and avert planned surgery to remove tonsils [82].

*P. grandiflorus* root extracts and *P. grandiflorus* saponin components have been developed by researchers for their antiviral activity. This formula can be used effectively as a preventive or a therapeutic agent for hepatitis C. Moreover, used in clinical practice, this formula is harmless to humans [83].

In addition, apart from the clinical applications mentioned previously, the bioactive components of *P. grandiflorus* also have several other applications. However, chemical methods inevitably produce side-reactions and environmental pollution [84]. There is little research evaluating enzymatic preparation methods to modify triterpene saponins of *P. grandiflorus*. Wie et al. used *Aspergillus niger* crude enzyme extract to transform *P. grandiflorus* to many partially degraded glycosides. This confirms that biotransformation of *P. grandiflorus* has the potential of high efficiency and low toxicity [85].

### 4.4. Other Applications

**Figure 7:**
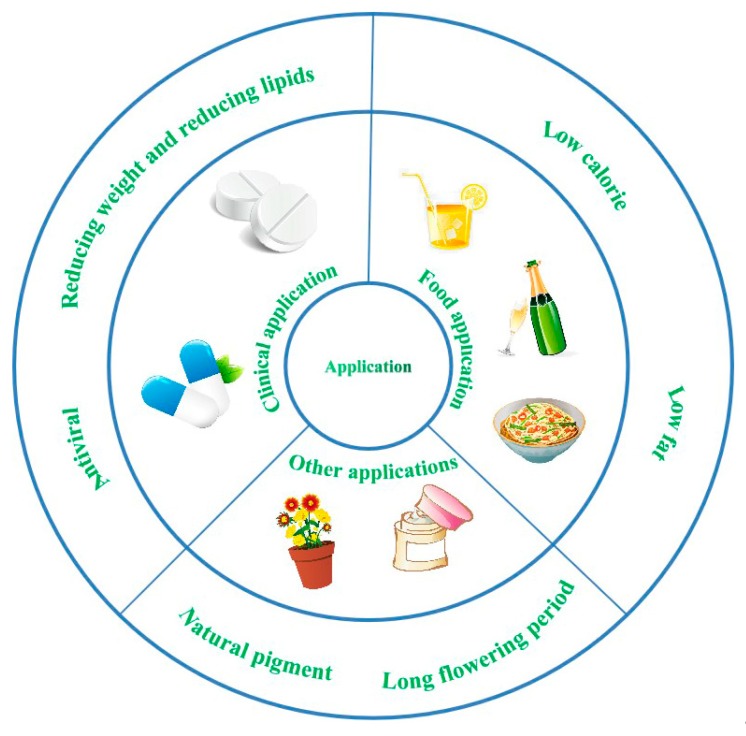
The application of *P. grandiflorus.*

*P. grandiflorus* contains stable anthocyanins, hence it also has the characteristics of a natural pigment. *P. grandiflorus* can thus be used as a natural food pigment in foods and beverages while playing a certain role in regulating physiological functions in the human body. The extract of *P. grandiflorus* has antioxidant effects and eliminates oxygen free radicals. It can be used in the development of anti-oxidation and anti-aging cosmetics. At the same time, the preparation of flavors and pigments from *P. grandiflorus* by acid electrolysis can be used in the production of cosmetics. Recent studies also show that the saponin of *P. grandiflorus* can affect the lipid content in the serum and liver, which has the effect of reducing weight and lipids. Moreover, *P. grandiflorus* has a long flowering period and the colorful flowers are widely used in flower baskets and bouquets [86]. The application of *P. grandiflorus* illustrative figure is shown in Figure 7.

## 5. Conclusions

*P. grandiflorus* is a good resource of health food, mainly including proteins, amino acids, trace elements, vitamins, and other substances. In recent years, with the continuous enhancement of people’s pursuit of nutritious and healthy food and increasing health care awareness, *P. grandiflorus* has been developed as a medicinal health supplement, functional food, and cosmetic component, especially in the dietary aspect. However, further studies need to be performed to make optimal use of this herb.

At present, studies on *P. grandiflorus* mainly focus on its chemical constituents and pharmacological activities. Modern medical research shows that *P. grandiflorus* has beneficial lung function, clears the throat, moistens the skin, and has preventive effects on respiratory tract diseases. It can also reduce blood pressure and promote blood circulation. *P. grandiflorus* can also regulate the intestines and stomach, promote digestion, and aid in respiratory tract infections. Overall, *P. grandiflorus* has great development potential as a medicine and food homologous variety.

The production process of this herb will also have an impact on its nutritional value. Pickled vegetables are a traditional fermented food in Korea, and its production process determines the quality of the product. The health aspects of pickled vegetables of *P. grandiflorus* are mainly attributed to the saponins and polysaccharides. Some studies have shown that vitamin C, vitamin B, carotene, and the trace element selenium in *P. grandiflorus* can block the synthesis of nitrite and nitrosamine, thereby reducing the content of nitrite. In order to ensure that the properties of *P. grandiflorus* are retained, the existing production process should be optimized to avoid the loss of nutrients during processing.

The pharmacodynamic basis and mechanisms of *P. grandiflorus* are not very clear, and there is a lack of epidemiological investigation data and clinical trials assessing the clinical effects of this herb. Therefore, further systematic and in-depth research is needed. At the same time, as more applications have been found in recent years, the natural resources of wild *P. grandiflorus* can no longer meet the needs. Therefore, it is necessary to strengthen research on cultivation measures to improve the quality of *P. grandiflorus* and promote the development of the industry. These studies will provide new ideas for the development of better therapeutic drugs and health products.

## Figures and Tables

**Table 1:** The other flavonoids components isolated from *P. grandiflorus.*

No.	Name	Ref.
1	Platyconin	[36]
2	Apigenin	[36]
3	(2R,3R)-taxifolin	[36]
4	Luteolin	[36]
5	Quercetin-7-*O*-glucoside	[36,38]
6	Quercetin-7-*O*-rutinoside	[36,38]
7	Platycoside	[36,39,40]
8	Delphinidin-3-rutinoside-7-glucoside	[36,41]
9	Flavoplatycoside	[36]

**Table 2:** The others components isolated from *P. grandiflorus.*

Classes	No.	Compound Name	Ref.
**Phenolic acids**	1	Caffeic acid	[42]
	2	3,4-dimethoxycinnamic acid	[42]
	3	Ferulic acid	[42]
	4	Isoferulic acid	[42]
	5	*m*-coumaric acid	[42]
	6	*p*-coumaric acid	[42]
	7	*p*-hydroxybenzoic acid	[42]
	8	*α*-resorcylic acid	[42]
	9	2,3-dihydroxybenzoic acid	[42]
	10	2-hydroxy-4-methoxybenzoic acid	[42]
	11	Homovanillic acid	[42]
	12	Chlorogenic acid	[42]
	13	Iobetyol	[43]
	14	Iobetyolin	[43]
**Polyacetylene**	15	lobetyolinin	[44]
	16	Lobetyolin	[41]
	17	Platetyolin A	[36,49]
	18	Platetyolin B	[36,49]
**Sterols**	19	Betulin	[41]
	20	*β*-sitosterol	[41]
	21	*δ*-7-stigmastenone-3	[27]
	22	Spinasterol	[36]
	23	*α*-spinasteryl-3-*O*-*β*-D-glucoside	[36]
**Others**	24	Threonine	[36]
	25	Valine	[36]
	26	Phenylalanine	[36]
	27	Methionine	[36]
	28	Isoleucine	[36]
	29	Leucine	[36]
	30	Lysine	[36]
	31	Inulin	[36]
	32	Grandoside	[36]
